# Emerging Evidence on Membrane Estrogen Receptors as Novel Therapeutic Targets for Central Nervous System Pathologies

**DOI:** 10.3390/ijms24044043

**Published:** 2023-02-17

**Authors:** Agnieszka Wnuk, Karolina Przepiórska, Bernadeta Angelika Pietrzak, Małgorzata Kajta

**Affiliations:** Laboratory of Neuropharmacology and Epigenetics, Department of Pharmacology, Maj Institute of Pharmacology, Polish Academy of Sciences, Smetna Street 12, 31-343 Krakow, Poland

**Keywords:** membrane estrogen receptors, rapid estrogen signaling, nervous system, neuroprotection, stroke, Alzheimer’s disease, mER

## Abstract

Nuclear- and membrane-initiated estrogen signaling cooperate to orchestrate the pleiotropic effects of estrogens. Classical estrogen receptors (ERs) act transcriptionally and govern the vast majority of hormonal effects, whereas membrane ERs (mERs) enable acute modulation of estrogenic signaling and have recently been shown to exert strong neuroprotective capacity without the negative side effects associated with nuclear ER activity. In recent years, GPER1 was the most extensively characterized mER. Despite triggering neuroprotective effects, cognitive improvements, and vascular protective effects and maintaining metabolic homeostasis, GPER1 has become the subject of controversy, particularly due to its participation in tumorigenesis. This is why interest has recently turned toward non-GPER-dependent mERs, namely, mERα and mERβ. According to available data, non-GPER-dependent mERs elicit protective effects against brain damage, synaptic plasticity impairment, memory and cognitive dysfunctions, metabolic imbalance, and vascular insufficiency. We postulate that these properties are emerging platforms for designing new therapeutics that may be used in the treatment of stroke and neurodegenerative diseases. Since mERs have the ability to interfere with noncoding RNAs and to regulate the translational status of brain tissue by affecting histones, non-GPER-dependent mERs appear to be attractive targets for modern pharmacotherapy for nervous system diseases.

## 1. Background

Estrogens is a general term that refers to steroid hormones such as estrone (E1), 17β-estradiol (E2), estriol (E3), and estetrol (E4). Among them, the most potent and prevalent is E2. Estrogens are mainly synthesized by the ovaries and adrenal glands and, to a lesser extent, by adipose and breast stromal tissues and the brain, acting on multiple receptors to regulate gene transcription, neural function, and behavior. Additionally, some exogenous compounds, such as naturally derived phytoestrogens (e.g., genistein, daidzein) and synthetic xenoestrogens (e.g., bisphenol A, benzophenone-3), possess properties similar to those of estrogens that may disturb the hormonal cellular response [1,2,3,4].

These steroids not only cause transcriptional effects over the course of hours to days, but they are also locally synthesized within neural circuits to rapidly (within minutes) modulate a range of behaviors, such as spatial cognition and communication. The biological effects of estrogens are mediated by two major subtypes of estrogen receptors (ERs)—nuclear (classical) ERs and nonnuclear (nonclassical, membrane, extranuclear) ERs. Both types of ERs are widely distributed throughout tissues and organs, including the central nervous system. Classical ERs regulate the majority of hormonal effects on reproductive and adipose tissues, whereas mERs enable acute regulation of estrogenic signaling, particularly in the cardiovascular, metabolic, and nervous systems. Nuclear estrogen receptors (nERs) include ERα/ESR1 and ERβ/ESR2, which act as transcription factors and become membrane-associated through a post-translational modification called palmitoylation. The membrane-embedded ERs are located mainly in caveolae, which can act as scaffolds for signaling molecules. Apart from membrane-associated ERα (mERα) and ERβ (mERβ), the group of nonnuclear estrogen receptors (mERs) includes GPER1 (GPR30), ER-X, and Gq-mER, all of which act via second messengers to enable the acute regulation of estrogenic signaling throughout the organism.

Nuclear- and membrane-initiated estrogen signaling cooperate to orchestrate the pleiotropic effects of estrogens. It is generally accepted that ER-mediated signaling plays key roles in neurogenesis [5,6,7], neurodevelopment [8], and neuroprotection [9,10,11], and its impairment may lead to a predisposition to neurodegenerative diseases [12,13] and mood disorders [14]. However, the application of estrogens as neuroprotectants in humans presents numerous limitations, mainly due to detrimental side effects triggered by the activation of classical ERs in peripheral tissues. For this reason, in recent years mERs have emerged as potential therapeutic targets for inducing neuroprotection, avoiding the hormonal side effects elicited by the activation of classical nuclear ERs.

The aim of this review is to present the unique signaling and properties of nonnuclear ERs, with particular emphasis on mERα and mERβ, and their therapeutic capacities in the treatment of nervous system disorders. Since metabolic dysfunctions increase the risks of Alzheimer’s disease and stroke, the effects of mERs on metabolic homeostasis, vasculature, and pain propagation are presented in addition to mER-mediated neuroprotection and cognitive improvements.

## 2. Interactions with the Cell Membrane

**Figure 1:**
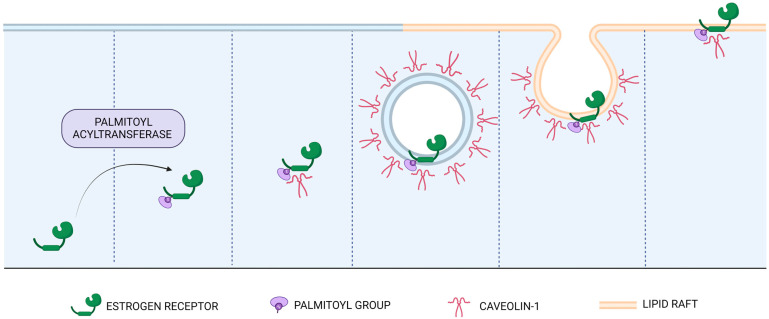
Membrane ERα/β (mERα/β) interaction with the cell membrane. The shuttling of estrogen receptors to the membrane depends on the addition of a palmitoyl group to Cys447 by palmitoyl acyltransferase. Palmitoylation facilitates the physical interaction of mERα/β with caveolins in the caveolae, cell membrane lipid-rich rafts known as signalosomes.

Interactions with the cell membrane are essential for the functioning of the membrane fraction of ERs. In addition to the nuclear localization of the majority of ERs, some are also present in the plasma membrane, endoplasmic reticulum, and mitochondria. A pool of 5–10% of the classical estrogen receptors ERα and ERβ is located in the cell membrane, where they are embedded in lipid rafts [15,16]. The process of shuttling ERs to the membrane depends on their post-translational modification, which consists of the addition of a palmitoyl group to Cys447 by palmitoyl acyltransferase [17,18,19]. Palmitoylation facilitates the physical interaction of both mERα and mERβ with the membrane protein caveolins in the caveolae, cell membrane lipid-rich rafts known as signalosomes [20,21]. The process has been visualized in Figure 1. Malfunctions of mER-signalosome complexes have been suggested to trigger Alzheimer’s disease pathology [22]. The palmitoylation process is essential for mER-caveolin interactions, and mutation of the ERα palmitoylation site prevents this interaction [18]. A mutant mouse in which the *Zdhhc7* gene encoding the palmitoyl acyltransferase is constitutively inactivated presents an interesting model for the study of ER palmitoylation and the functions of membrane-associated ERs. It is important to know that there are several subtypes of mERα in the cell membrane: in addition to full-length ERα66 (66 kDa), splice isoforms of ERα, e.g., ERα36 (36 kDa) and ERα46 (46 kDa), are also transmembrane proteins [23].

A completely different type of membrane estrogen receptors are the G-protein-dependent estrogen receptors (GPERs), such as GPER1 (previously known as GPR30), Gq-coupled mER (Gq-mER), and ER-X, whose distinguishing feature is a multipass transmembrane domain that anchors receptors to the plasma membrane. During translation, G-protein-coupled receptors are inserted into the membrane of the endoplasmic reticulum and then pass through the Golgi apparatus to eventually be transported to the cell membrane [24].

Some mERs present distinct subcellular distributions: mERα is mainly expressed in endolysosomes, mERβ prevails in mitochondria, and the majority of GPER1 is in the plasma membrane (breast cancer cells), endoplasmic reticulum, or Golgi apparatus (hippocampus, cortex, striatum) [25,26]. In particular, mERs are present in cortico-limbic brain areas; e.g., ER-X is mainly localized in the neonatal hippocampus and neocortex, but it was recently also found in the adult brain after ischemic stroke [27]. Neuronal function can be rapidly modulated by mERs; however, the putative functions of the receptors are only partially recognized. In the neonatal hippocampus, no sex differences were detected in the gene expression of *Cav-1* and *Zdhhc7*, which allow ERα and ERβ to associate with the plasma membrane. Interestingly, in the adult hippocampus, these specific genes showed decreased expression in females compared to males [28].

## 3. Signaling Pathways of Membrane ERs vs. Nuclear ERs

ER localization imposes the involvement of particular signaling pathways in the functioning of nuclear and mERs. The action of nuclear ERs relies mainly on releasing from Hsp70/Hsp90 complexes, receptors dimerization, translocation to the nucleus, and evoking transcriptional effects. A small proportion of ERα and ERβ (approx. 10%) interacts with plasma-membrane-associated signaling proteins to activate intracellular signaling cascades and to exert rapid nontranscriptional/nongenomic effects that ultimately alter transcriptional responses [29,30]. Similarly, G-protein-dependent estrogen receptors mediate many rapid responses to estrogen in the brain, particularly in hippocampal neurons, where GPER1 modulates second messenger signaling via Gs- and Gi/o-associated increases in cAMP and PI3K or Src [31].

The genomic mechanism of the action of ERs relies on the regulation of gene expression through the ligand-induced activation of ERs or by phosphorylation. ERs dissociate from chaperone proteins (Hsp70/Hsp90) and dimerize, forming homo- or heterodimers that are translocated into the nucleus. This is followed by the recognition of the promoter region known as the estrogen response element (ERE) [32]. Approximately 30% of ER-regulated genes do not contain the ERE sequence, but their transcription is targeted by transcription factors such as SP-1 or AP-1. Different isoforms of ERα and ERβ result from alternative splicing of specific genes. Apart from ligand-dependent activation, ERα is known to be activated in a ligand-independent way, namely, through EGF or IGF-1 [33]. ERβ forms homodimers or heterodimers with ERα. The receptor has tumor-suppressive and antiproliferative properties that compete with the functions of ERα in the reproductive system [34].

Post-translational modification of sex steroid receptors can direct them to extranuclear sites, such as the plasma membrane, where the steroid receptors may initiate rapid signaling. Classical nuclear ERs, i.e., ERα and ERβ, are devoid of transcriptional activity when located on the cell membrane [35]. mERα and mERβ have internal and C-terminal splice variants and undergo N-terminal truncations. The truncated proteins may associate with caveolin to initiate signaling on the plasma membrane [36]. The covalent attachment of palmitic acid (palmitoylation) to target proteins such as ERs predisposes the receptors to coupling with membrane microdomains and mediating rapid, nongenomic effects [37]. In addition, the receptors are organized by caveolin proteins into microdomains with mGluRs to elicit functional signaling [28].

Instead of genomic effects, membrane-anchored ERs exert rapid actions through cytoplasmic signal transduction cascades. mERα- and mERβ-mediated rapid estrogen signaling is focused on the activation of protein kinases, mainly protein kinase B (PKB or Akt) and mitogen-activated protein kinases (MAPKs) [38]. The activation of the receptor causes its interaction with the p85 regulatory subunit of phosphatidylinositol 3-kinase (PI3K), which then phosphorylates and activates Akt, localizing it in the plasma membrane [39]. This conserved kinase cascade is now considered to be responsible for E2-dependent effects on cell proliferation and cell migration [40]. The second leading pathway induced by estrogens is mediated by MAPKs, including extracellular-activated kinases (ERKs), which are responsible for cell growth, proliferation and differentiation, as well as p38/MAPK and c-Jun N-terminal kinases (JNKs), which are activated in response to cellular stress [41]. Additionally, key molecules such as cyclic adenosine monophosphate (cAMP) and endothelial nitric oxide synthase (eNOS) are generated in response to E2 stimulation and a rapid rise in intracellular calcium levels (Ca^2+^) [42]. Evidence suggests that membrane-initiated estrogen signaling (MIES) may upregulate nitric oxide (NO) production through the PI3K and MAPK pathways [43].

A different mechanism of mER action is interacting with the Gα protein family and transmitting signals through the adenylyl cyclase (AC)-cyclic adenosine monophosphate (cAMP)- dependent protein kinase A (PKA) cascade, phospholipase C cascade and Ras homologous (Rho) protein cascade. Through these signaling pathways, mERs are involved in the cell migration that is essential during neuronal remodeling, wound healing, angiogenesis, and atherogenesis as well as cardiovascular protection, by regulating endothelial H_2_S release [25,44,45]. The activation of GPERs results in coupling to Gi, Go and Gs proteins and leads to the activation of kinase pathways, including the PI3K and MAPK pathways, as well as Ca^2+^ mobilization and cAMP synthesis, not directly but through the transactivation of the epidermal growth factor receptor (EGFR). The mechanism of GPER1 action also involves other signaling pathways, such as NOTCH, insulin-like growth factor receptor (IGFR), nuclear factor kappa-light-chain-enhancer of activated B cells (NF-κB), and Hippo/Yes-associated protein (Hippo/YAP) [38].

**Figure 2:**
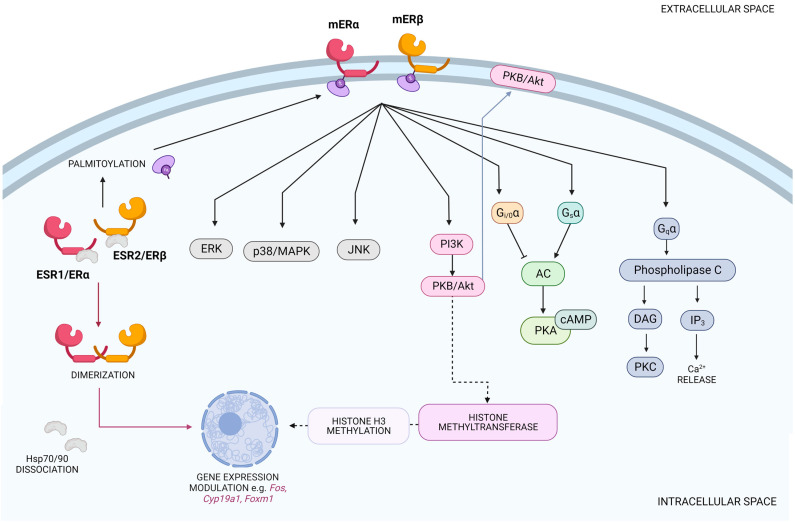
Main signaling pathways initiated by membrane ERα and membrane ERβ activation. Rapid estrogen signaling is commonly mediated via MAPKs, Akt, and G proteins. ER—estrogen receptor, PKA—protein kinase A, PKB/Akt—protein kinase B, PKC—protein kinase C, ERK—extracellular signal-regulated kinase, DAG—diacylglycerol, CREB—cAMP response element-binding protein, PI3K—phosphoinositide 3-kinase, IP_3_—inositol trisphosphate.

Interestingly, rapid responses to external stimuli via mERs can initiate the transcription of diverse estrogen-regulated genes. It has been shown that the increased PI3K/Akt signaling pathway regulates the activity of epigenetic-related enzymes such as histone methyltransferases through interaction with the polycomb repressive complex 2 (PRC2) and the MLL/complex of proteins associated with Set I (COMPASS). The role of PRC2 in silencing gene expression consists of adding methyl groups at Lys27 of histone H3 to form H3K27me3, a commonly known marker of gene repression [46,47]. Additionally, mERα and mERβ can indirectly modulate gene expression by changing the activation of the potent transcription factor CREB as a result of cAMP activity [48]. Like non-GPER-dependent mERs, GPERs can regulate gene expression through CREB but also through the oncogene C-FOS [49,50,51]. Under conditions of hypoxia, GPER1 interacts with hypoxia inducible factor-1α (HIF-1α) to activate genes that code for connective tissue growth factor (CTGF), vascular endothelial growth factor (VEGF), and interleukin 6 (IL-6), among others. Unfortunately, by interfering with cellular dynamics, GPER1 becomes involved in the development and progression of different types of cancer [38]. An overview of mER signaling pathways can be found in Figure 2.

## 4. Localization of mERs in the Nervous System

ERs are widely expressed throughout the brain, acting as transcriptional effectors or rapidly initiating membrane and cytoplasmic signaling cascades. In addition to the brain, ERα is present in the reproductive and musculoskeletal systems and the heart, where it regulates development and functioning. Apart from the brain, ERβ is mainly localized in the female reproductive system, but it also plays a tumor-suppressive role in a variety of tissues [52]. The majority of mERs are expressed in the reproductive, metabolic, cardiovascular, and nervous systems of organisms. Several studies have shown that mERs are present throughout the central nervous system, particularly in the prefrontal cortex, dorsal striatum, nucleus accumbens (NAc), and hippocampus, where they are responsible for the rapid effects of estrogen involved in, for example, learning and memory processes [53,54]. ER-X, which is localized in the mouse brain (hippocampus, neocortex) and uterus, mainly during the first postnatal month, has been suggested to participates in the neuronal differentiation of the cerebral cortex and early sexual development [52].

Full-length ERα is a 66 kDa protein, and ERα Δ4, which lacks exon 4, is 52 kDa. Both versions of ERα are highly expressed as membrane-associated fractions. At the plasma membrane, mERα associates with the metabotropic glutamate receptor mGluR1a, whereas mERα Δ4 binds to mGluR2/3, as detected in the rat arcuate nucleus [55]. The interactions of mERs and mGluRs engage caveolin proteins. Caveolin 1 is responsible for the interactions of mERα with mGluR1 or mGluR5, and this transactivation leads to the activation of MAPK/cAMP, which results in Ca^2+^ release. The interactions of caveolin 3 with mERα or mERβ are associated with mGluR2 and mGluR3 when cAMP and Ca^2+^ flux is reduced [56,57,58,59].

In the hippocampus, ERα-mGluR1a interactions activate endocannabinoid signaling, which subsequently decreases GABA neurotransmission [60]. Similarly, the activation of ERα by E2 attenuates the GABAergic postsynaptic response. However, the activation of Gq-mER by STX has been shown to enhance the GABAergic postsynaptic response in NPY/AgRP neurons, since it enhances the coupling of GABA_B_ receptors to GIRK channels [61]. In the NAc, ERs are present mainly at extranuclear sites, specifically as membrane-associated receptors localized primarily on axons and axon terminals, both catecholaminergic and GABAergic. Only a small proportion of the receptors (approx. 10%) are on NAc glial cells. This could explain the rapid effects of estrogens on dopamine release and receptor availability, suggesting that mERs interfere with or regulate neurotransmission presynaptically [62].

Male mice with impaired mERα signaling exhibit increased numbers of kisspeptin- and calbindin-immunoreactive neurons in the anteroventral periventricular nucleus of the hypothalamus. This effect does not occur in females with impaired mERα signaling. This suggests that mERα receptors are responsible for the development of these neuronal populations only in males [63]. In female postpubertal hypothalamic astrocytes, mERα-mGluR1a activation through E2 induces an increase in Ca^2+^ concentration, which is necessary for progesterone synthesis and can be blocked by the mGluR1a antagonist LY 367385 [64]. In the ventrolateral division of the hypothalamic ventromedial nucleus, membrane-initiated estrogen stimulation can induce progesterone receptor expression [65]. Striatal brain regions such as the caudate, putamen, and NAc, which mediate critical behavioral functions (e.g., motivated behavior, reward, learning, and sensorimotor function), have been found to express membrane-associated estrogen receptors in a sex-specific manner [66]. GPER1 is expressed in both the peripheral and central nervous systems, e.g., in the striatum, pituitary, hippocampus, substantia nigra, brainstem, and several nuclei of the hypothalamus [67]. In the hypothalamus, GPER1 has been recognized to regulate serotonin receptor signaling and gonadal functions [68,69,70]. As for Gq-mER, it has been shown that its activation influences energy homeostasis, thermoregulation, and reproductive behaviors as well as gonadotropin and corticotropin-releasing hormones [71].

## 5. Neuroprotection Mediated by Membrane ERs

Although the majority of estrogen-induced neuroprotection has been attributed to classical nuclear ERs, there is growing evidence for the neuroprotective potential of nonnuclear ERs. Initially, researchers focused on GPER1, which became a main focus in studying the neuroprotective capacity of phytoestrogens (e.g., ginsenoside Rg1), flavonoids (geinstein, daidzein), and synthetic SERMs (e.g., STX) [1,2,11,72]. Selective activation of GPER1 appeared to cause neuroprotection in animal models of mood disorders, Alzheimer’s disease, and Parkinson’s disease, but there is no consensus on the role played by GPER1 in ischemic stroke [73]. This is probably the reason that interest was recently directed toward non-GPER-dependent nonnuclear ERs, namely, mERα and mERβ. Additionally, no serious controversies have been recognized with respect to the actions mediated by mERα and mERβ in neuronal tissue [74].

### 5.1. GPER-Mediated Neuroprotection

Previously, we showed the key involvement of GPER1 in the neuroprotective action of daidzein against glutamate-induced excitotoxicity, as evidenced by upregulated receptor expression and a loss of neuroprotection in *Gper1*-silenced cells [2]. We also showed that apoptosis of mouse embryonic neuronal cells induced by the pesticide DDT or the biocide triclocarban was accompanied by an impairment of GPER1 signaling that supported the neuroprotective property of the receptor and was verified by the use of selective ligands, specific siRNA, and electrophysiology [75,76]. Furthermore, the triclocarban-induced decrease in GPER1 expression corresponded to the hypermethylation of the *Gper1* gene. Our discoveries are in line with the neuroprotective effect of STX, a synthetic SERM that activates GPER1 and Gq-mER without other mERs, normalizing ATP content and mitochondrial gene expression and recovering the outgrowth of dendrites in Tg2576 neurons that was decreased due to exposure to amyloid-β (Aβ) [77]. However, in contrast to the neuroprotective capacity of GPER1 identified as being involved in daidzein or STX activity and suppressed in response to DDT or triclocarban, the treatment of mouse neurons with the UV filter benzophenone-3 led to a stimulation of GPER1 signaling in terms of *Gper1* gene hypomethylation and enhanced mRNA and protein expression levels, suggesting the neurotoxic potential of the receptor [78].

As mentioned, the role of GPER1 in protecting brain neurons from hypoxia and ischemia remains controversial. It has been suggested that GPER1-mediated neuroprotection relies on the inhibition of apoptosis and excessive autophagy via the modulation of the PI3K-Akt-mTOR signaling pathway [79]. Since the role of autophagy in hypoxia/ischemia-induced brain pathology is ambiguous, the involvement of GPER1 in neuroprotection or neurotoxicity may be a result of interference with the autophagy process that determines the stroke duration and damage area. The latest study by Pemberton et al. (2022) shed light on the differential effects of GPER1 depending on brain structure. In rat embryonic cerebral tissue, the effects of GPER1 activation by a selective G1 agonist (e.g., neural growth, neural firing activity) were more profound in hippocampal neurons than in cortical neurons, independent of the higher GPER1 expression in the cortex compared to the hippocampus [26]. The lack of an obvious or inverse correlation between the GPER1 expression level and specific neuroprotective effects suggests that other mechanisms are involved, such as the stimulation of neurogenesis, the inhibition of neuroinflammation, or the modulation of nuclear ER-mediated effects [80,81,82].

The neuroprotective property of GPER1 is supported by a study showing that selective activation of GPER1 by G1, or of GPER1 and Gq-mER by STX, attenuated hippocampal neuron loss in the CA1 region when the compounds were administered to ovariectomized middle-aged female rats immediately after global ischemia [83]. The alleviation of ischemic injury in ovariectomized female mice that were subjected to middle cerebral artery occlusion (MCAO) has been explained by GPER1-dependent inhibition of TLR4-mediated microglial inflammation [82]. Recently, GPER1 was suggested to inhibit neuroinflammation in rat cerebral ischemia by regulating microglial phenotypes [84]. A new GPER1 agonist, CIFTA, was found to increase axonal and dendritic outgrowth in the rat embryonic hippocampus [85]. Intriguingly, selective activation of GPER1 by G1 appeared to increase the infarct volume in male mice subjected to cerebral ischemia, which was accompanied by elevated expression of active caspase-3 in peri-infarct neurons [86]. Furthermore, GPER1 distribution was increased in the peri-infarct brain regions of male mice, but was decreased in the infarct core of both sexes of mice subjected to ischemia–reperfusion [87]. In our study, there was no evidence of GPER1-mediated neuroprotection against hypoxia-induced damage in response to raloxifene [88]. Similarly, Roque et al. (2019) did not observe any effect of GPER1 activation on OGD-exposed primary cortical neurons. The only GPER1-induced effect was directed at astrocyte death due to the rise in intracellular calcium levels during ischemia [73].

### 5.2. mERα- and mERβ-Mediated Neuroprotection

In contrast to GPER1, much less is known about the neuroprotective properties of non-GPER-dependent mERs. It has been suggested that estrogens may preserve the brain through a dual membrane-related mechanism, namely, by interaction with ER-related signalosomes to protect against neurotoxic insults and by lipostatic action to preserve lipid balance in neuronal membrane microdomains. Estrogens exert neuroprotective effects against Alzheimer’s disease partly by binding to ERs embedded in signalosomes, which are preferentially located in lipid rafts, i.e., dynamic membrane microstructures characterized by a peculiar lipid composition enriched in gangliosides, saturated fatty acids, cholesterol, and sphingolipids. It has been shown that the reduction in E2 blood levels occurring during menopause induced a disruption of ER-signalosomes in frontal cortical brain areas. Similar ER-signalosome derangement was observed in brains from Alzheimer’s disease patients [89].

In the cortex and hippocampus, mERα, along with caveolin 1, cooperates with important cellular mediators such as insulin growth factor-1 receptor beta (IGF-IRβ) and a voltage-dependent anion channel (VDAC) [90,91,92]. A decrease in mER-signalosome complexes and insufficient mER-VDAC phosphorylation in the frontal cortex during menopause have been connected with cognitive deficits and the promotion of Aβ toxicity [22]. mERα-dependent inhibition of the apoptosis regulator DAXX protein translocation and the upregulation of VDAC phosphorylation are postulated to trigger Alzheimer’s disease [93,94,95]. The activation of mERα by the nonclassical estradiol pathway activator estren (4-estren-3α, 17β-diol) restored Aβ-induced learning deficits and the loss of cholinergic cortical projections mainly through direct phosphorylation of MAPK/CREB in basal forebrain cholinergic neurons and restored cholinergic fibers [96]. We have also shown neuroprotection in the Aβ-based model of Alzheimer’s disease following selective activation of mERs, specifically mERα and mERβ, by Pathway Preferential Estrogen-1 (PaPE-1) [97]. The exposure of mouse primary neurons to PaPE-1 inhibited Aβ-evoked neurotoxicity, oxidative stress, and apoptosis, particularly the mitochondrial apoptotic pathway, as evidenced by the normalized mitochondrial membrane potential and restored BAX/BCL2 ratio.

PaPE-1 ((S)-5-(4-hydroxy-3,5-dimethyl-phenyl)-indan-1-ol) was designed by Madak-Erdogan et al. in 2016 to provide beneficial metabolic and vascular effects without stimulating reproductive tissues. PaPE-1 interacts with the mER signaling pathway without activating the nuclear pathway or the controversial GPER1 [98]. PaPE-1 administered before inducing stroke in female mice (MCAO model) attenuated neuroinflammation and caused long-term improvement in motor coordination without undesirable uterotrophic or cancerogenic effects [99]. Pretreatment with PaPE-1 (subcutaneous pellets) reduced the brain infarct volume and leukocyte infiltration into the ischemic brain in mice subjected to MCAO. Our recent publication provided a “proof of concept” confirming the ability of posttreatment with PaPE-1 to provide neuroprotection against hypoxic and ischemic injuries (a 6 h delay of initiation of hypoxia/ischemia) [74]. The mechanism of PaPE-1 neuroprotection relied on inhibition of apoptosis and ROS formation as well as restoration of cellular metabolic activity.

Regarding specific mERα subtypes, a newly identified ERα36 that mediates nongenomic estrogen signaling via MAPK/ERK is highly expressed in the central nervous system and actively involved in neuroprotection [100]. It was shown that ERα36-mediated rapid estrogen signaling protects PC12 cells from OGD-induced damage [54]. The ERα36-attributed action also relied on an inhibition of H_2_O_2_-induced oxidative stress in human SH-SY5Y and IMR-32 cells, which was abrogated by the knockdown of the receptor [101]. Decreased or knocked-down expression of the ERα36 gene resulted in increased GSK3β and BAX expression, elevated tau protein levels, and enhanced susceptibility of human neuroblastoma SH-SY5Y cells to apoptosis [16,102]. In addition to mERα- and/or mERβ-mediated neuroprotective effects against Aβ toxicity (Alzheimer’s disease) and hypoxic/ischemic damage (stroke), the expression of mERα, but not mERβ, was upregulated in MPP+-exposed SH-SY5Y cells. This might be a protective mechanism against MPP+ that maintains cell homeostasis through the upregulation of the MAPK/ERK signaling pathway and the promotion of autophagy maturation in an in vitro model of Parkinson’s disease [103].

**Figure 3:**
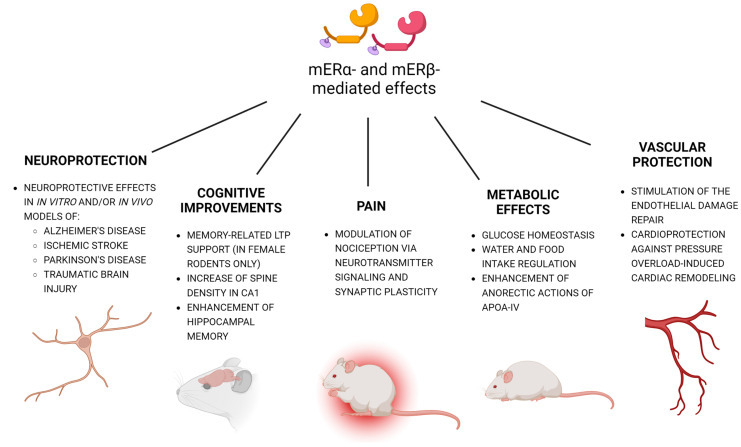
Membrane ERα/β (mERα/β)function throughout the body. In addition to facilitating neuroprotection, mERα/β improve memory and cognition as well as affect social behavior. mERα/β activation also modulates pain and influences metabolic and vascular systems.

For the E2-bovine serum albumin (BSA) conjugate (a membrane-impermeant analog of 17β-estradiol), the activation of the membrane fraction of ERs with E2-BSA evoked neuroprotective effects against traumatic brain injury, as evidenced by inhibited brain edema, blood-brain barrier (BBB) integrity maintenance, and decreased intracranial pressure [35]. E2-BSA inhibited traumatic cerebral-contusion-induced apoptosis through the activation of ERK1/2 and Akt [104]. Moreover, E2-BSA decreased ischemic neuronal injuries and preserved cognitive function in the Morris water mazetest by activating the ERK-Akt-CREB-BDNF signaling pathway [105]. Although E2 and E2-BSA protected hippocampal neurons from ischemic damage, E2-BSA was less effective than E2 [106]. Treatment with E2-BSA also appeared to be neuroprotective against chronic minimal peroxide stress by lowering apoptosis rates and upregulating the autophagic pathway by increasing ERK phosphorylation and decreasing p-p38 levels [107]. Stress was found to upregulate mERα expression on the surface of SH-SY5Y cells, which has been associated with the expression of the MAP2 cytoskeletal protein, known to be involved in microtubule assembly and neurogenesis. However, one should be cautious when interpreting evidence concerning the effects of E2-BSA conjugates because even freshly prepared E2-BSA solutions contain free E2. Furthermore, since E2 is linked to BSA through sites necessary for ER binding, E2-BSA binds poorly to the ER. In addition, the biological activity of the conjugates can be impaired because of the high molecular weight, which probably results from high protein cross-linking during the conjugation reaction [108,109,110]. A summary of mERα and mERβ function throughout the body is illustrated in Figure 3.

## 6. Cognitive Improvements and Pain

In addition to facilitating neuroprotection, mERs improve memory and cognition and affect social behavior and pain. Again, most of the data refer to GPER1-mediated effects, particularly with respect to mood disorders, Alzheimer’s disease, and traumatic brain injury, which have been studied in rodent models. It has been shown that treatment with G1 protects against traumatic brain injury, which is considered a risk factor for Alzheimer’s disease, and improves early-onset cognitive impairment in rats via the PI3K/Akt pathway [111]. Similarly, GPER1 activation inhibited oxidative stress, neuroinflammation, and apoptosis, improving memory and cognitive dysfunctions in a mouse model of Alzheimer’s disease (5XFAD), a mouse model of vascular dementia, and a mouse model of 3,5-dihydroxyphenylglycine (DHPG)-induced long-term depression (LTD) [68,72,112]. Evidence has shown that a decrease in GPER1 expression impairs learning and memory, which involves actin depolymerization and the SRC-1 and PI3K/mTORC2 pathways [113]. In hippocampal field CA1, a region critical for spatial learning, the activation of GPER1 increased dendritic spine density and hippocampal memory consolidation [114]. Dendritic spine remodeling underlies not only learning and memory but also social behaviors, including social recognition, social learning, social preference and responses, and agonistic behaviors [115]. In this context, GPER1 has been suggested to act as a modulator of ERα-mediated action, e.g., by targeting spinogenesis and driving female social learning and social behaviors such as lordosis [116]. Moreover, in the paraventricular nucleus of the rat hypothalamus, the desensitization of 5-HT1A serotonin receptors that is necessary for SSRI therapeutic efficacy depends on GPER1 [117].

Only sparse data refer to the involvement of non-GPER-dependent mERs in regulating cognitive function and behavior. It has already been mentioned that activating mERα may restore not only the Aβ-induced loss of cholinergic fibers but also Aβ-induced learning deficits [96]. The receptors mERα and mERβ have been detected in dendritic spines [118], where they rapidly mediate estrogen signals by increasing spine density within 30–60 min and enhancing spatial memory within 2–4 h [119]. mER-dependent modulation of synaptic plasticity and NMDAR/AMPAR transmission has been supported by the colocalization of E2-BSA-FITC with GluN1 and GluA1 in spinal dorsal horn neurons [120]. In contrast to males, female rodents have higher synaptic levels of ERα and require mERα for the activation of signaling kinases of Src, ERK1/2, and postsynaptic TrkB that support long-term potentiation (LTP) and learning [102]. These differences explain why men are generally superior to women in remembering spatial relationships. It has been suggested that social behaviors such as social investigation, preference, recognition, and memory, as well as anxiety-related behaviors in the social context, are regulated by E2 via not only nuclear but also mERs. It has been shown that the activation of mERα and mGluR1a leads to the stimulation of the µ-opioid receptor (MOR) in the arcuate nucleus and medial preoptic nucleus, leading to female sexual receptivity [121,122].

Women appear more sensitive to chronic pain disorders than men. One of the possible mechanisms relies on mER-mediated attenuation of thermal antinociception that engages nociceptin/orphanin FQ peptide (NOP) receptors [123]. Following spared nerve injury, selective mER activation by E2-BSA or G1 inhibited an NOP-mediated antinociceptive effect in rats in an antagonist-reversible manner. This is in line with abolishing opioid receptor-like 1 (ORL1)-mediated spinal antinociception in response to the activation of GPER1 or Gq-mER in male and ovariectomizedfemale rats [124]. Furthermore, GPER1 present in the rostral ventromedial medulla appeared to contribute to the chronification of postoperative pain (thermal and mechanical) in rodents [125]. According to the latest optogenetic study of Jiao et al. (2022), bulbospinal GABAergic-ON neurons specifically express GPER1, which drives pain and morphine tolerance when activated [126]. Regarding non-GPER mERs, during diestrus, mERα/mGluR1 signaling appeared to suppress spinal endomorphin 2 analgesia [127]. Similarly, in diestrus, mERα-mediated activation of mGluR1 suppressed spinal endomorphin 2 antinociception. However, in proestrus, spinal endomorphin 2 antinociception was facilitated independently of mERα, namely, through glutamate-activated mGluR1 [128].

## 7. Metabolic Effects and Vascular Protection

Since metabolic and vascular abnormalities increase the risks of stroke and Alzheimer’s disease, the relevant information showing the impact of mERs is important in the context of central nervous system pathologies.

GPER1 has recently been postulated as a novel therapeutic target for treating obesity and comorbid metabolic dysfunctions on the basis of murine models showing that the selective GPER1 agonist G1 limited weight gain, reduced insulin resistance, and improved glucose and lipid homeostasis [129]. Similar effects were observed in ovariectomized female mice, i.e., in a model of postmenopausal obesity, where treatment with G1, in addition to reducing body weight and improving glucose homeostasis, increased energy expenditure and lowered body fat content [130]. Additionally, experiments on GPER1 KO mice pointed to regulatory functions of GPERs on body weight and metabolism [131]. According to Davis et al. (2014), male GPER1 KO mice developed moderate obesity at 8 weeks of age, whereas female GPER1 KO mice developed increased body weight 6 weeks later than males [132]. In both sexes, significant reductions in energy expenditure were also observed, thus confirming a key role of GPER1 in the development of postpubertal energy balance.

Glucose homeostasis is maintained by estrogens primarily via nuclear ERα, which mediates protective effects against type 2 diabetes and obesity. Experiments on transgenic mERα (MOER; membrane-only ERα) mice showed fasting and fed hyperglycemia as well as glucose intolerance in both sexes [133]. Since the activation of mERα in the medial preoptic area appeared to decrease overnight food and water intake in ovariectomized female rats, mERs have been postulated to control ingestive behaviors that occur at least in part through interactions with mGluR [134]. This is in line with PaPE-1-induced activation of mERα/mERβ, which reduced body weight gain and fat accumulation in the adipose tissues (perigonadal, perirenal, and subcutaneous) of ovariectomized mice [98] and significantly decreased liver weight and lipid accumulation in diet-induced obesity or leptin-deficient obese mice [135]. In these last two models, PaPE-1 also lowered the expression of genes associated with fatty acid metabolism and collagen deposition [98,135]. Moreover, it has been shown that the activation of mERα along with the PI3K/Akt pathway enhances the anorectic action of apolipoprotein A-IV (apoA-IV), as evidenced by significantly increased Akt phosphorylation in response to E2-BSA [136]. In addition to the estrogen-dependent protection against obesity in animals fed ad libitum, mERα was found to contribute to energy homeostasis by promoting eating in hungry mice [137].

Both GPER and membrane-associated non-GPER ERs were found to promote vasorelaxation, which relies on an increase in nitric oxide (NO) production due to eNOS phosphorylation, RhoA-associated kinase (ROCK) inhibition, and/or ERK/ GSK3β signaling activation [138]. Through these pathways, mERs are postulated to reduce cardiac remodeling and the progression of myocardial infarction-induced heart failure. In a recent study, Ma et al. (2022) demonstrated an improvement in the cardiovascular function of aging female rats after prolonged activation of GPER1 with G1 combined with inhibition of nuclear ER signaling [139]. Similarly, Yu et al. (2022) observed great improvement in aortic relaxation in young adult and middle-aged female rats, but not in male rats [140]. The results are in line with increased GPER1 expression and GPER1-mediated vasorelaxation in rat uterine arteries during gestation [141]. In contrast, GPER1 deletion appeared to significantly reduce endothelial function in adult female rodents [142] and to increase the size of atherosclerotic lesions in female mice [143]. Regarding vascular effects preferably mediated by non-GPER mERs, activating mERα/mERβ by PaPE-1 was found to stimulate endothelial repair in mice subjected to ovariectomy [98]. Moreover, experiments on transgenic mice lacking ERα nonnuclear signaling, i.e., KRRKI/KI (KRR knock-in mutant ERα) mice, showed that the missing signaling played a pivotal role in cardioprotection against pressure-overload-induced cardiac remodeling and was essential to the therapeutic efficacy of cGMP-PDE5is (cGMP-phosphodiesterase 5 inhibitors) in the treatment of heart failure [144].

## 8. Controversies about GPER1

As mentioned, GPER1 is the most extensively characterized GPER-dependent membrane ER. In recent years, the receptor was studied as a mediator of the beneficial effects of estrogens that has potent therapeutic capacity and, in contrast to the nuclear ERs, is devoid of severe, mainly hormonal, side effects. Despite triggering neuroprotective effects, cognitive improvements, and vascular protective effects and maintaining metabolic homeostasis, GPER1 has become the subject of controversy. One concern is related to the ambiguous role of GPER1 activation during cerebral hypoxic or ischemic injury and the recently recognized inhibitory action of activated GPER1 on the proliferation of neural stem/progenitor cells [145]. Another concern regards the involvement of GPER1 in the neurotoxic effects of environmental pollutants (e.g., benzophenone-3) and in pain propagation [146,147]. However, the most serious problem is GPER1 participation in malignant tissue development [148]. Importantly, aberrant GPER1 expression can be used to predict cancer progression and poor survival in patients with breast and gynecological cancers [149,150]. Since GPER1 contributes to a microenvironment that is conducive to tumor development and progression, GPER1 signaling could represent a promising target in anticancer therapy; however, in this regard, the receptor has to be blocked/antagonized.

The increased risk of tumorigenesis is the most serious contraindication for therapeutic use of GPER1-directed ligands in the treatment of central nervous system diseases. Since mERα and mERβ can be selectively targeted by PaPE-1, which has a safe pharmacological profile, in our opinion searching for selective ligands of the receptors will yield better prospects for designing future pharmacotherapies of brain pathologies.

## 9. Perspectives

In contrast to GPER1, concern has not been raised about the involvement of non-GPER-dependent mERs, i.e., mERα and mERβ, in tumorigenesis. Additionally, there is no evidence of their negative impact on neural stem cell proliferation or differentiation. Except for participation in pain propagation, the activation of non-GPER-dependent mERs elicits beneficial effects. These include protective effects against brain damage, synaptic plasticity impairment, memory and cognitive dysfunctions, metabolic imbalance, and vascular insufficiency. We postulate that highly selective activation of specific mERs is an emerging platform for the development of a novel ER-targeted therapy that is rapid and devoid of the negative, mainly hormonal, side effects associated with nuclear ERs.

Recent studies have shown that dietary long-chain polyunsaturated fatty acid (LCPUFA) supplementation improves spatial and recognition memory in aging female mice [151]. During aging, brain lipid composition changes affect lipid raft integrity and may impair memory function. Since lipid rafts are important elements for mERα/mERβ-mediated signaling, activating the receptors may cause even stronger therapeutic effects than diet supplementation alone. Additionally, the localization of mERβ and/or mERα within the mitochondrial membrane provides unique regulatory mechanisms that are essential for cellular homeostasis; thus, strategies directed at membrane-associated ERs may play causative roles in preventing the neural degeneration that is triggered by energy loss, e.g., in Parkinson’s disease [9,10,11].

It is generally accepted that metabolic syndrome and type 2 diabetes increase the risks of Alzheimer’s disease and related dementia, as well as stroke and thromboembolic episodes [152,153,154]. Recognized pathomechanisms of Alzheimer’s disease revealed ERs as exemplary targets for therapeutics that could control the production and neurotoxicity of Aβ peptide. Furthermore, 17α-estradiol-induced stimulation of dendritic spine density in hippocampal neurons exposed to HIV-1 gp120 appeared to depend on endolysosome localization of ERα [155]. Therefore, selective targeting of mERα and/or mERβ may lead to the development of novel therapies and open up new therapeutic perspectives for Alzheimer’s disease-related pathologies, including therapies to improve cognition and synaptic function. Since ER signaling deficiency leads to a predisposition to stroke and thromboembolism, particularly in postmenopausal women, targeting mERα and mERβ may restore impaired signaling and protect the mammalian brain from hypoxic/ischemic injuries [156,157]. 

Although ERs are known to control neuroinflammation, which is a common feature of nervous system disorders, the roles of membrane-associated non-GPER-dependent ERs in shaping microglia or synthesizing cytokines need to be determined. In a mouse model of allergic encephalomyelitis (chronic neuroinflammation), reactive astrocytes express mainly ERα and, to a lesser extent, ERβ, but infiltrated leukocytes express ERs in their membranes [158]. Since *Esr1* gene dysfunction was shown to trigger neuroinflammation [159], one may assume that the dysfunction partially affects mERα, suggesting its anti-inflammatory potential.

Recent identification of a functional link between ERs, both nuclear and membrane, and noncoding RNAs provides prospects for targeting mERs with specific lncRNAs or miRNAs to treat ER-related pathologies, including nervous system disorders. However, only sparse data point to non-GPER mERs as regulators of the translational status of brain tissue. It has been shown that early exposure to estrogen leads to mERα-mediated activation of the MLL4/COMPASS methyltransferase, which trimethylates histone 3 at lysine 4 (H3k4me3) to activate transcription. mERα-mediated acetylation of histone 4 (H4K5ac) is also postulated [47]. mERs also appear to regulate the histone methyltransferase enhancer of zeste homolog 2 (EZH2), which reduces the trimethylation of lysine 27 on histone H3 [160]. Finally, the reverse correlation between ERα36 and pro-angiogenic miR210 suggests the therapeutic potential of mERs [161].

## Data Availability

Not applicable.

## Abbreviations

Aβ—amyloid β; BSA—bovine serum albumin; E1—estrone; E2—estradiol; 17β-estradiol; E3—estriol; E4—estetrol; ERE—estrogen response element; ER—estrogen receptor; ERα/ESR1—estrogen receptor α; ERβ/ESR2—estrogen receptor β; GPER1/GPR30—G protein-coupled estrogen receptor 1; GPER—G protein-coupled estrogen receptor; Gq-mER—Gq-coupled membrane estrogen receptor; mER—non-classical, membrane, extra-nuclear estrogen receptors; OGD—oxygen and glucose deprivation; ROS—reactive oxygen species; SERM—selective estrogen receptor modulator; TBI—traumatic brain injury.
